# The distribution of rennet activity between the cheese aging process and whey is not influenced by the association of enzymes with caseins

**DOI:** 10.1016/j.heliyon.2024.e32263

**Published:** 2024-05-31

**Authors:** Seyed Mehrdad Mirsalami, Mahsa Mirsalami, Afshar Alihosseini, Amin Ghodousian

**Affiliations:** a Department of Chemical Engineering, Faculty of Engineering, Islamic Azad University Central Tehran Branch, Tehran, Iran; b Faculty of Engineering Sciences, Raja University, Qazvin, Iran; c Department of Engineering Science, University of Tehran, Tehran, Iran

**Keywords:** Rennet activity partitioning, Rennet curd, Rennet whey, Calf rennet, Milk concentration

## Abstract

The division of rennet in cheesemaking is split between the curd and whey, influencing the taste and texture of aged cheeses. Our study aimed to examine how raising the protein concentration in reconstituted skim milk (up to 8.8 %) affects the distribution of calf rennet activity (RA) in rennet curds produced through two methods: renting only and renneting with glucono-δ-lactone (GDL) to achieve slow acidification. The distribution of rennet activity (RA) into curds increased as the concentration of skim milk rose, ranging from 8.6 % to 29.1 % without acidification, and from 6.5 % to 19.4 % when combined with slow acidification. This increase seemed to be related to the retention of moisture and protein. Surprisingly, the concentration of residual RA in the whey (measured in international milk clotting units, IMCU/mL) remained unaffected and remained consistent with the initial IMCU/mL of milk. This suggests that the division of RA between curd and whey is not influenced by the association of enzymes with caseins (CNs). Instead, it is possible that the strength of interactions between CNs themselves plays a significant role. These findings could be valuable for research focused on enhancing the cheese aging process.

## Highlights

• Increasing skim milk concentration affects rennet distribution in cheese aging.
• Rennet activity in curds increases with higher skim milk concentration.
• Retention of rennet activity in whey remains consistent despite milk changes.
• The association of enzymes with caseins does not influence rennet division.
• Interactions between caseins play a significant role in rennet distribution.

## 1 Introduction

The gelation of milk is a crucial stage in the production of most cheeses. This step involves using one or a mix of coagulants, such as clotting enzymes and acids, to disrupt the stability of caseins (CNs), which exist naturally in milk as stable colloidal dispersions [1]. Calf rennet, which has been utilized for cheesemaking for a long time, consists of milk clotting enzymes. Commercial rennets, depending on the age and diet of the calf, may contain 45–80 % chymosin, an enzyme that breaks a specific bond in κ-CN, leading to the aggregation of CN micelles and the clotting of milk [2,3].

The clotting activity or strength of calf rennet is determined by the concentration and purity of chymosin. However, since it also contains pepsin, which has different proteolytic and clotting activities compared to chymosin, rennet activity (RA) is preferably measured in terms of individual activities (% or mg/L) [4]. Milk clotting assays are used to quantitatively assess RA by establishing the time it takes for milk to clot under specific conditions, with rennet concentration and clotting time exhibiting a linear inverse relationship [5]. Previously, standard conditions for these assays involved a Berridge substrate consisting of 9 % reconstituted skim milk in a 0.01 N CaCl_2_ solution, with a renneting temperature of 37 °C. However, newly proposed international standard conditions for total RA assessment include a 0.1 % (v/w) CaCl_2_, pH 6.8, a temperature of 35 °C, and reference rennet powders with 2000 IMCU/g [6].

Similar to the components present in milk, the rennet activity (RA) utilized for milk coagulation in cheesemaking is divided between curd and whey. If the RA is not denatured by the cheesemaking process, the portion that is retained remains active [7]. This active fraction plays a significant role in the proteolytic action, particularly on αs-CN, which contributes to the development of flavor and texture in aged cheeses. To determine the partitioning of RA (whether it is retained in the cheese or lost in the whey), a common method involves employing a synthetic heptapeptide in an RP-HPLC assay [8,9].

The cheesemaking process relies on the crucial adjustment of salt balance, which exerts a significant impact on the coagulation of casein micelles by rennet. The initial stage of cheesemaking is recognized to be influenced by factors such as pH, calcium value, protein composition, and the calcium bound to casein [10]. The gelation kinetics of rennet in reverse osmosis (RO) extracts exhibited a decelerated pattern, characterized by prolonged rennet coagulation time and reduced maximal firming rate when compared to Ultrafiltration (UF) concentrates [11]. The gelation characteristics and firmness of the curd have an impact on the ultimate output and composition of cheese. Previous studies have demonstrated that the firmness of the gel during cutting influences not only the moisture content of the cheese but also the recovery of fat and protein. There have been endeavors to manufacture cheeses using milk condensed through RO. Altay et al. [9] conducted a study where Cheddar cheese was produced using whole milk that was concentrated by a factor of three. The resulting cheese, made from RO-condensed milk, exhibited a composition similar to that of conventional Cheddar cheese. However, it displayed an elevated lactose amount and a heterogeneous grainy consistency [12]. The yield and formation of cheeses produced from RO condensed milks containing 9%–13 % solids concentration were investigated by Bansal et al. [13]. In their research, the authors noted a higher lactose concentration in the cheese made from RO milk with a solid concentration of 13 % compared to the control cheese. Additionally, they observed a cheese yield increase of 1–2.5 % above the estimated yields. In spite of the favorable effect on yields, elevated lactose levels in cheese can cause quality issues during aging as a result of post-acidification occurrences [12]. Consequently, the utilization of RA milk may be constrained to cheeses with a brief maturation period or ought to be confined to low amount ratios. In a previous study by Düsterhöft and colleagues [14], an examination was conducted on milk essences with a solids content of 20 %, specifically focusing on their cheesemaking properties. It was observed that when using RO milk, model cheeses displayed a greater yield when adjusted for moisture, as well as a higher moisture level when compared to cheeses produced from skim milk as the control.

Theoretically, approximately 2–5% of the total rennet activity (RA) would be retained in the cheese curd. However, empirical values exhibit significant variation depending on factors such as the type of cheese, rennet used, and milk concentration [15,16]. For instance, reported values include 5 % retention in pressed Cheddar cheese, 42 % retention in Camembert cheese, and 85 % retention in Feta cheese made from ultrafiltered milk. In previous study, observed that approximately 72 % of the total RA added to milk was found in a mixture of fresh drain and pressed rennet whey (simulated through centrifugation) [17]. Additionally, the concentration of RA in the whey (measured in international milk clotting units per milliliter of whey) was equivalent to the initial concentration in the milk (measured in international milk clotting units per milliliter of milk). The objective of our present study was to investigate whether similar findings could be observed when there are changes in milk concentration. We aimed to uncover the factors that drive the partitioning of RA during milk renneting under two conditions: rennet only and rennet combined with slow acidification [18,19].

## 2 Materials and methods

### 2.1 The creation of reconstituted skim milk samples

**Fig. 1:**
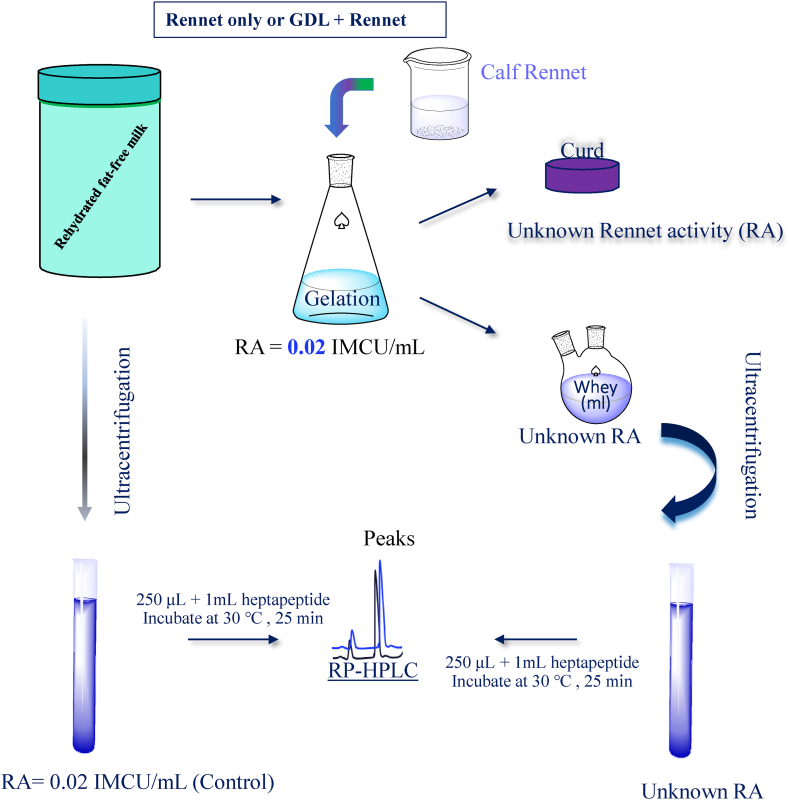
Experimental design.

The experimental setup for this study is depicted in Fig. 1. To create reconstituted skim milk samples with varying protein content, low-heat skim milk powder (obtained from Warrnambool Cheese and Butter) was utilized. Pre-determined quantities of the skim milk powder were gradually introduced into 1 L of Milli-Q water contained in a beaker positioned on a magnetic stirrer. The mixture was continuously stirred at room temperature for a duration of 2 h, following which the reconstituted skim milk samples were refrigerated overnight to ensure complete protein hydration.

### 2.2 The coagulation of reconstituted skim milk samples using rennet

Each reconstituted skim milk sample was divided into two sets of 100 mL. In the first set, coagulation was achieved through renneting under slow acidification using GDL. The GDL was adjusted to attain a drain whey pH of 6.5. The second set was coagulated solely by renneting. To ensure uniformity, all samples were pre-warmed to a renneting temperature of 35 °C using a water bath. Coagulation was initiated by adding 0.01 IMCU (International Milk Clotting Units) of freshly diluted calf rennet solutions (10 × dilution, 310 IMCU/mL, sourced from Pak.Co, Tehran, Iran). The samples containing GDL exhibited faster setting times and were coagulated for 25 min. On the other hand, the samples without GDL had slower setting times and were coagulated for 1 h. Subsequently, all coagulated samples were cut, cooked at 36 °C for 45 min, and the whey was separated by centrifugation at 2200×*g* using an Merck 3346R centrifuge (manufactured by Merck, CA, USA).

### 2.3 Analysis of protein and moisture content

To determine the total protein (T-Protein) content of each sample, triplicate measurements were conducted using an ultrasonic milk analyzer (manufactured by LACTOSCAN, Midland Park, New Jersey, USA). The methodology used for protein determination followed the procedure outlined in a previous study by John K. Towns [1]. On the other hand, the moisture content was assessed by subjecting 25 g of each sample to overnight drying at 120 °C, following the method described by Nina Kröncke [3].

### 2.4 Measuring the residual rennet activity (RA) in the whey and determining its distribution between the curds

The concentration of residual rennet activity (RA) in the rennet whey samples was determined using a RP-HPLC method. This method was developed by Joseph F. Kayihura [20], with a modified procedure described by M.J Hurley [17,21]. As described in previous research, a portion of each milk sample used for rennet coagulation and its corresponding whey sample were subjected to duplicate ultra-centrifugation. This ultra-centrifugation was carried out at 120,000×*g* and 30 °C for a duration of 40 min, utilizing a Fisher Ultra FU-30 type centrifuge (manufactured by Thermo Fisher Scientific, CA, USA). To serve as controls, a known amount of RA was added to the supernatants obtained from the skim milk samples (0.01 IMCU/mL of supernatant). To conduct the experiment, 250 μL aliquots of both the controls and their corresponding whey samples were individually combined with a 1 mL solution of a synthetic heptapeptide (Pro–Thr–Glu–Phe–[NO_2_–Phe]–Arg–Leu) obtained from Mimotopes (located in Tehran, Iran). All the solutions were pre-incubated at 30 °C. The mixtures were thoroughly mixed using vortexing and then incubated at 30 °C for a duration of 30 min. After the incubation period, the reaction was halted by adding 30 μL of a pepstatin solution obtained from Sigma-Aldrich Pty. Ltd (based in Macquarie Park). It is important to note that the timing of various steps, including pre-incubation, reaction time, addition of pepstatin, and vortexing [22], is crucial for accurate results in this experiment. Even a slight delay of a few seconds can have a significant impact on the outcomes.

Following the addition of pepstatin, the samples were promptly subjected to vortexing, followed by filtration through a 0.55 μm filter. Subsequently, the filtrates were analyzed using RP-HPLC. The analysis was conducted using specific conditions: an AV-2200C, Zenith-X system manufactured by Sony Corporation (located in Tokyo, Japan), a C18 column (Titan® 5 mm, 280 Å, 230 × 15 mm, obtained from Agilent Technologies based in Santa Clara, California, USA), and an oven temperature of 20 °C. The UV-detector from Shimadzu was set to 250 nm. The mobile phase consisted of two components: A, which was composed of 0.2 % trifluoroacetic acid (TFA, sourced from Sigma Aldrich) in Milli-Q water, and B, which was composed of 0.2 % TFA in 86 % acetonitrile (Sigma Aldrich). The flow rate was set to 0.5 mL/min, and the elution gradient followed a specific pattern: 2 min at 20 % B, followed by a gradual increase to 70.5 % B over 20 min, and finally 20 min at 20 % B. The measurements were performed in triplicates, and the estimation of the unknown residual rennet activity (RA) in the whey was determined based on peak ratios using the equation [1] provided below:

[1] (PAc/PAw)=(0.02/RAw)

where *PAc* represent the peak area of the hydrolysate ([NO_2_–Phe]–Arg–Leu) generated from the control sample, which involves the known addition of RA to the milk supernatant. Similarly, let *PAw* denote the peak area of the hydrolysate produced by the unknown RA in the rennet whey. The control sample had a known concentration of 0.01 IMCU/mL of RA. On the other hand, the whey sample contained an unknown concentration of RA, represented by *RAw* in IMCU/mL.

Subsequently, the percentage of RA that partitioned into the curds was determined by calculating the difference between the initial total RA added to the milk and the total RA found in the whey. This difference was then divided by the initial total RA added to the milk and multiplied by 100 to obtain the percentage. The total RA in the milk or whey was obtained by multiplying their respective RA concentrations (expressed in IMCU/mL) by their respective total volumes (measured in mL).

### 2.5 Statistical analysis

The experiment was replicated three times, ensuring a minimum of two analyses for each repetition. The data was evaluated using a split-plot design. Analysis of variance was conducted on the experimental measurements, followed by Tukey's honestly significant difference test, both performed with a confidence level of 95 %. The statistical analyses were executed using the software package (SAS Institute Inc., Cary, NC, USA). Significance was determined at a threshold of P < 0.05.

## 3 Results and discussion

### 3.1 Investigating the hydrolysis of a heptapeptide by calf rennet under different conditions

**Fig. 2:**
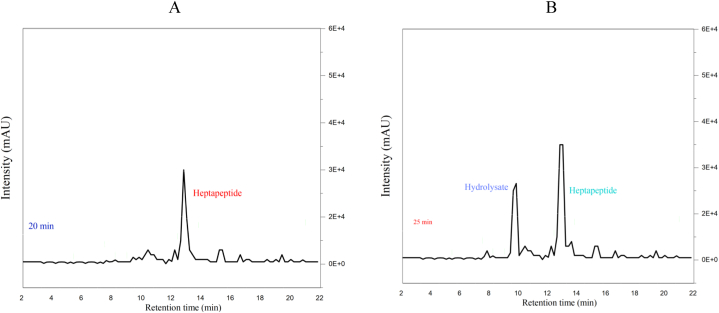
Displays two typical chromatograms: (A) the initial heptapeptide (Pro–Thr–Glu–Phe–[NO_2_–Phe]–Arg–Leu) and (B) the heptapeptide residue and the resulting hydrolysate ([NO_2_–Phe]–Arg–Leu) after incubation at 30 °C for 25 min with the ultracentrifugal supernatant of reconstituted skim milk containing 0.02 International Milk Clotting Units (IMCU) of calf rennet. Glucono-δ-lactone (GDL) and rennet activity (RA) are denoted abbreviations in this context.

**Fig. 3:**
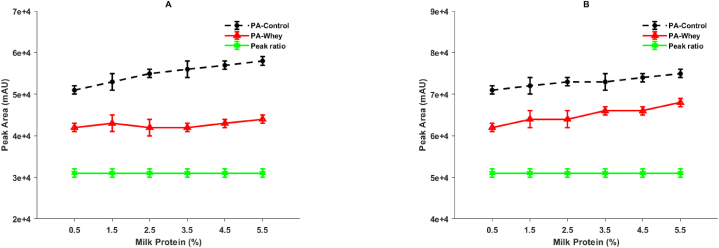
Illustrates the alterations in peak areas of the hydrolysate of a synthetic heptapeptide caused by the action of calf rennet, which was introduced into ultracentrifugal supernatants (peak area-control) or recovered in whey (peak area-whey). Additionally, the concentration of residual rennet activity (RA) in terms of International Milk Clotting Units (IMCU) was estimated in the whey obtained from reconstituted skim milk with varying protein concentrations. Panel (A) represents samples that were treated with glucono-δ-lactone (GDL), whereas panel (B) portrays samples without GDL.

Gaining insight into the partitioning of RA during the cheesemaking process is crucial for effectively managing the quality of both aged cheeses and whey-derived products. This is particularly important as proteolysis is desirable in aged cheeses but not in whey-derived products. To investigate the partitioning of calf RA in freshly produced curds obtained from reconstituted skim milk with varying concentrations, the hydrolytic activity of the unknown RA recovered in the whey was compared to that of a known control RA on a synthetic heptapeptide. In Fig. 2, it can be observed that there is a distinct single peak representing the initial heptapeptide (Fig. 2A), as well as its reduction and the resulting hydrolysate (Fig. 2B) after 30 min of rennet action at the Phe_4_– [NO_2_ – Phe_5_] bond. The elution profile of the heptapeptide and its hydrolytic product remains consistent with the original method described by Bin Li [6]. However, certain modifications were made to the procedure, including changes in column type and size, solvent concentration, and elution gradient, resulting in shorter retention times. The changes in peak areas of the hydrolysate, as depicted in Fig. 3, serve as an indicator of the hydrolytic activity of calf rennet under different milk concentrations and two pH levels. Specifically, pH 6.5 was used for acidified samples prepared with GDL (Fig. 3A), while natural pH conditions were maintained for the other samples (Fig. 3B). Notably, the peak areas show an increase as the milk concentration used for both the control and rennet whey rises. However, it is important to highlight that the values obtained under acidification (Fig. 3A) differ from those obtained without acidification (Fig. 3B). This observation aligns with the findings of Pier Giorgio Righetti [23], who also reported a decrease in peak areas of the hydrolysate resulting from acidification.

These findings suggest that the hydrolytic activity of calf rennet on the heptapeptide is influenced by both the concentration of the sample and the pH conditions. These differences underscore the importance of preparing standards (control samples) under similar conditions to those of the test samples (whey). Similar considerations apply when conducting milk clotting assays Leite Júnior [15], since the concentration of the sample, as well as renneting conditions such as pH and temperature, can impact the aggregation rate of CNs, which in turn affects the milk clotting time. Additionally, the types and concentrations of enzymes and additives, such as CaCl_2_ or NaCl, along with the milk concentration and renneting conditions, have been shown to have an impact on the rate of rennet action on κ-CN [6,24]. Thus, whether determining RA through milk clotting or RP-HPLC assays, and performing estimations using calibration curves or peak ratios, it is advisable to prepare the standards or controls using serums obtained from the same milk used to prepare the rennet whey.

### 3.2 Assessing the partitioning of rennet activity (RA), moisture, and total protein into the rennet curd and evaluating the curd yield

**Fig. 4:**
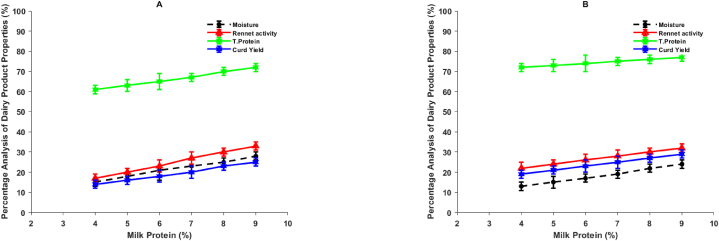
Alterations in the yields of fresh curd and the retention of total protein (T-Protein), moisture, and rennet activity (RA) were observed in rennet curds manufactured from skim milk that had been reconstituted with varying levels of protein concentration. (A): samples containing glucono-δ-lactone (GDL), and (B): samples lacking GDL.

Fig. 4A illustrates the changes in RA, moisture, and T-protein partitioning into rennet curds, specifically for samples renneted under acidification. Conversely, Fig. 4B showcases the same changes but for samples renneted without acidification. A notable observation across all samples is the linear increase in both RA, moisture, and T-protein retentions as the milk concentration increases. As expected, the increase in curd yield is directly associated with the retention of both moisture and protein. Furthermore, it is evident that as the concentration of reconstituted skim milk rises, the retention of RA in the curds also increases in proportion to the retention of moisture and T-protein. Under the combined process of renneting and slow acidification, the partitioning of RA into curds exhibited an increase from 6.5 % of the total RA added to milk with 4 % protein, to 19.4 % of the total RA added to milk with 8.8 % protein. It is worth noting that the RA partitioning observed at 3 % milk protein aligns closely with the theoretical value of 6–7% for the final RA retention in cheese curds [23], and is similar to the reported 6 % retention in pressed Cheddar cheese [25]. In the absence of acidification, a similar pattern was observed, but with higher values (8.6–29.1 %), as there was less moisture expelled. The RA retention values discovered in this study for curds derived from milk samples containing 2.5–7% protein, through the process of renneting and acidification, fall within the range of 5–17 % reported for typical Cheddar cheese [23]. These values are also close to the range of 8–15 % reported for miniature Cheddar cheese produced from whole milk with 4 % protein, as well as 14.5–19.5 RA units/kg of Cheddar cheese [18].

Furthermore, the findings of this study are consistent with the observations made by Xiaohui Huang [10], who noted higher levels of residual RA in high moisture cheeses. They also attributed the increase in RA due to milk concentration to limited whey drainage. The relationship between RA and moisture aligns with the results reported for Cheddar cheeses with variations in calcium, phosphorus, and lactose content [17]. However, it is important to note that Flavio Tidona [5] reported a higher retention of 25 % RA in Cheddar cheese made from whole milk, which exceeds the values observed in the present study for samples produced under acidification, as well as the ranges reported in other studies as mentioned earlier. M. Ozturk attributed these variations within the same cheese variety to slight differences in the applied manufacturing protocols [3].

However, the findings presented in this study offer a contrasting perspective. Previous research has established a direct relationship between the concentration of the hydrolysate derived from the synthetic heptapeptide and RA through calf rennet [23]. Hence, in this study, the peak ratios were considered as equivalent to RA ratios, as previously described. Interestingly, regardless of the skim milk concentration and acidification, the peak ratios of the unknown to the control RA remained constant (Fig. 3). Since the controls contained the same RA, a consistent peak ratio implies that the concentration in the test samples was also consistent. This observation is further supported by the results presented in Table 1, which demonstrate that the concentration of residual RA in rennet whey (IMCU/mL) did not vary with either the reconstituted skim milk concentration or the acidification process. These findings are consistent with our previous study and align with the work of Maria De Angelis [7], who reported no change in residual RA per kilogram of protein, as well as Abdelmoneim [25], who found no variation in the quantity of coagulant bound per gram of casein.

**Table 1:** Residual rennet activity (RA) concentration, measured in International Milk Clotting Units (IMCU), was evaluated in rennet whey derived from reconstituted skim milk samples with different protein concentrations. The evaluation was conducted under two conditions: combined renneting and slow acidification, achieved using glucono-δ-lactone (GDL), and renneting alone (without GDL).

Milk protein (%)	RA (IMCU/mL of whey)	
	With GDL	Without GDL
2.5	0.012 ± 0.002	0.011 ± 0.001
4	0.013 ± 0.002	0.013 ± 0.002
6.5	0.013 ± 0.004	0.013 ± 0.002
7	0.015 ± 0.005	0.015 ± 0.002
8.8	0.015 ± 0.006	0.015 ± 0.005

The information presented here, together with the observed linear relationship between RA retention and moisture retention (Fig. 4), as well as previous research indicating a direct correlation between total RA and moisture content in cheese provides compelling evidence that the retention of RA in rennet curds is not solely influenced by enzyme-casein associations. If this were the case, curds produced with acidification would have exhibited higher RA retention, as suggested by Ipek Altay [9]. The results obtained in this study, however, shed light on an alternative driving force: the strength of casein-casein interactions, which also govern the retention of moisture and other milk constituents, as well as curd yield. It should be noted that the specific mechanisms behind these casein-casein interactions require further investigation in future studies.

## 4 Conclusions

In conclusion, our study demonstrated that the distribution of rennet activity (RA) between curds and whey in the cheese aging process is not influenced by the association of enzymes with caseins. Instead, the strength of interactions between caseins themselves appears to play a significant role. The effect of increasing the concentration of reconstituted skim milk up to 8.8 % T-protein on the distribution of RA during renneting alone or combined with slow acidification achieved through GDL was assessed in the present investigation. The findings indicate that as the concentration of reconstituted skim milk rises, the retention of RA in curds increases proportionally with the retention of moisture, while the concentration of residual RA in whey (IMCU/mL of whey) remains unchanged. Therefore, the retention of RA in rennet curds is not influenced by the associations between the enzyme and CN. Rather, it is driven by the strength of CN–CN interactions, although the specific interactions' role remains unknown and necessitates further investigation in future studies. It is important to note that standards and test samples should be prepared under similar conditions, as the chemical solutions used can affect RA. This information could be valuable for research on accelerating the maturation process of concentrated milk systems in the production of aged hard cheeses. It would also be interesting to explore the retention of RA in rennet curds produced from other types of modified milk systems using different renneting conditions.

## Data availability statement

All data generated or analyzed during this study are included in this published article.

## Additional information

No additional information is available for this paper.

## Funding statement

This research did not receive any specific grant from funding agencies in the public, commercial, or not-for-profit sectors.

## CRediT authorship contribution statement

**Seyed Mehrdad Mirsalami:** Writing – review & editing, Writing – original draft, Visualization, Validation, Supervision, Software, Resources, Project administration, Methodology, Investigation, Funding acquisition, Formal analysis, Data curation, Conceptualization. **Mahsa Mirsalami:** Writing – review & editing, Resources, Project administration, Funding acquisition. **Afshar Alihosseini:** Supervision. **Amin Ghodousian:** Software.

## Declaration of competing interest

The authors declare that they have no known competing financial interests or personal relationships that could have appeared to influence the work reported in this paper.
